# Correlation genotype-phenotype: *MEFV* gene mutations and Moroccan patients with rheumatoid arthritis

**DOI:** 10.11604/pamj.2022.41.121.30368

**Published:** 2022-02-11

**Authors:** Hakima Missoum, Najlae Adadi, Mohammed Alami, Hamza Toufik, Abdelhakim Bouyahya, Fatima-Zahra Laarabi, Fatima Bachir, Abdellah El Maghraoui, Youssef Bakri

**Affiliations:** 1 Laboratory of Human Pathologies Biology, Department of Biology, Faculty of Sciences, and Genomic Center of Human Pathologies, Faculty of Medicine and Pharmacy, Mohammed V University in Rabat, Rabat, Morocco,; 2 Laboratory Autoimmunity, Department of Immunology, National Institute of Hygiene, Rabat, Morocco,; 3 Department of Medical Genetics, National Institute of Health, Rabat, Morocco,; 4 Laboratory of Microbiology and Molecular Biology, Faculty of Science, Mohammed V University, Rabat, Morocco,; 5 Rheumatology Department, Faculty of Medicine and Pharmacy, Military Hospital, Mohammed V University, Rabat, Morocco,; 6 Laboratory of Flow Cytometry, National Institute of Hygiene, Rabat, Morocco

**Keywords:** Rheumatoid arthritis, *MEFV* gene mutations, anti-citrullinated peptide antibodies, rheumatoid factor, autoimmune disease

## Abstract

**Introduction:**

rheumatoid arthritis (RA) is a systemic autoimmune disease primarily affecting the joints. Arthritic disorders are associated with mutations of the Mediterranean fever (MEFV) gene. The aim of this study is to show whether MEFV mutations will be involved in the pathogenesis of RA, to explore the frequency of these mutations and to study the genotype-phenotype correlation between mutations in this gene and a cohort of Moroccan patients with rheumatoid arthritis (RA).

**Methods:**

the present study included 100 patients with RA and 200 control group (CG) who were unrelated individuals from the same ethnic. All patients were tested for auto-antibodies: cyclic citrullinated peptide (ACPA/anti-CCP_2_), rheumatoid factor (RF) and were analyzed by Sanger Sequencing of the 2 and 10 exons of MEFV gene (hot-spot according to the literature).

**Results:**

we detected 13 missense variants already MEFV gene mutation reported in the literature (S154T, G222A, G230L, L611H, L695A, M694V, I720M, A737L, P758S, L709A, T732A, G687A and P743L). Carrier rates of MEFV gene mutations were 24/100 (24%) for the RA group and 4/200 (4%) for CG. In the RA group, we observed that no man has presented with MEFV mutation. In the RA group, while gender, BMI, RF and ACPA were significantly higher in the mutation carrier group than those of the non-carrier group (p<0.01). The level of C-reactive protein and HAQ were slightly elevated in the carrier group but not significant. No other significant differences were observed between patients with MEFV mutations and those without MEFV mutations.

**Conclusion:**

the results of this study suggest that MEFV gene mutations appear to be an aggravating factor severity of RA and consequently, patients with RA might be screened for MEFV gene mutations in countries where FMF is frequent. We report also that our study is the first one in our country Morocco.

## Introduction

Rheumatoid arthritis (RA) is a chronic inflammatory disease that affects about 0.5-1% of the population worldwide, resulting in more disability, joint damage, worsening of quality of life, and premature mortality in these patients than in general population [1]. The prevalence is estimated about 0.7% in Moroccan population (about 200,000 patients in Morocco) [2]. The incidence is highest between 40 and 60 years and women are 3 times more affected than men [3, 4].

Today, serological markers auto-antibodies rheumatoid factor (RF) [5] and anti-citrullinated protein/peptide antibodies (ACPA or anti-CCP) [6] allow the diagnosis and follow-up of the majority of patients with RA. Rheumatoid arthritis is a complex and multifaceted genetic disease that is influenced by both genetic and environmental factors which remain to be defined [7]. Genes that are known to be important for joint inflammation or the course of RA have been described primarily by the association of variations in genes encoding proteins. However, other genes different from human leukocyte antigens (HLA) were also tested, but the results were inconsistent [8]. Mediterranean fever (*MEFV*), suggesting that this gene could be involved in the pathogenesis of rheumatic diseases characterized by relapses of inflammatory episodes. This gene has already been identified as being responsible for familial Mediterranean fever (FMF).

Familial Mediterranean fever is caused by various mutations in the *MEFV* gene which is located on the short arm of chromosome 16p13.3 and comprises 10 exons [9], and this gene encodes a protein named pyrin/marenostrin consisting of 781 amino acids. These proteins are involved in innate immune responses and play a key role in the regulation of inflammasomal activity and apoptosis [10]. Furthermore, it has been reported that the presence of *MEFV* gene mutations might be a susceptibility factor for various inflammatory diseases [11], such as juvenile idiopathic arthritis (JIA) [12]. Moreover, it has also been revealed that *MEFV* gene mutations might be an aggravating factor for the severity of some inflammatory diseases including (RA) [13].

The aim of this study is to investigate whether the mutations of the *MEFV* gene are involved in the pathogenesis of RA. We adopted a case-control model to compare the frequency of *MEFV* mutation between RA patients and control group subjects and to compare the severity of disease between mutation carriers and non-carriers. This study is the first to explore the prevalence of *MEFV* gene mutations in Morocco.

## Methods

**Study population:** the study involved 100 RA Moroccan patients and 200 individuals for the control group (CG). Rheumatoid arthritis patients were recruited from the Rheumatology Department of Military Hospital Mohammed V (Rabat, Morocco) between April 2017 and December 2018. The criteria used for the clinical diagnosis of the RA disease are those described by the American College of Rheumatology (ACR)/European League Against Rheumatism (EULAR) for the classification of RA 2010 [14]. Only patients over 18 years of age were included. Exclusion criteria for RA patients: other types of inflammatory arthritis, including psoriatic arthritis, reactive arthritis, spondylarthropathies and inflammatory arthritis related to bowel disease. The control group (CG) eligible blood donors were recruited from the National Blood Transfusion Center and volunteered to take part in the study between February and December 2018. Only patients over 18 years of age were included. Exclusion criteria for CG must not have RA, autoimmune and/or inflammatory disease. Rheumatoid arthritis patients and CG were unrelated individuals from the same population.

The demographic and clinical data included age, gender, body mass index (BMI), inbred marriage, smoking, familial history, alcohol, depression, the duration of the evolution of RA. As well as factors related to the disease including erythrocyte sedimentation rate (ESR), C-reactive protein (CRP), disease activity score (DAS28 CRP) and health assessment questionnaire (HAQ). The treatment used for RA included oral corticosteroids, conventional synthetic anti-rheumatic drugs (DMARDs) (methotrexate, leflunomide and sulfasalazine) and biotherapies: synthetic biological anti-rheumatic drugs (bDMARDs): (rituximab, etanercep, influximab, tocilizumab). All subjects (patients and controls) in this cohort were tested for auto-antibodies cyclic citrullinated peptide (ACPA/anti-CCP_2_), rheumatoid factor (RF) and genetic analyses.

***MEFV* gene mutation analysis:** we collected blood samples from all our patients and the control group and their genomic deoxyribonucleic acid (DNA) was extracted from peripheral blood with ethylenediamine tetra acetic acid (EDTA) using the commercial Qiagen kit, following the manufacturer's instructions. The specific primers were synthesized by University of California Santa Cruz (UCSC) and are presented in Table 1. We performed a standard polymerase chain reaction (PCR) for all our patients by using the primers of exons 2 and 10 of the *MEFV* gene (Table 1). Polymerase chain reaction products were purified using ExoSAP and analyzed by standard Sanger dideoxy nucleotide sequencing using 3130 genetic analyzer (thermo fisher scientific). This research was carried out by the laboratory of genetic of NIH.

**Table 1:** PCR primer sequences (F: forward; R: reverse)

Primers	Forward	Reverse	Tm (°C)	Amplicon size (bp)
MEFV_Ex2.1	CTCCTCTGCCCTGAATCTTG	AAGGGCCTGCACTCCTTC	60	480
MEFV_Ex2.2	CAGGGCAAGCCTCGGAC	GGCCAGCCATTCTTTCTC	60	451
MEFV_Ex10.1	GAACCCTGTAGGGATGTTGC	CTCCTTTATTAGCAGGCGGG	60	406
MEFV_Ex10.2	CATCCATAAGCAGGAAAGGG	TTGAGTGTGAATGCAAGATACAAG	59	391

**MEFV:** Mediterranean fever, **Tm:** melting temperature

**Serums analysis:** serum parameters blood samples were taken from all of the subjects to detect the ACPA or anti-CCP_2_ was performed by ELISA (Bio-Rad laboratories, CA cutoff value 5 UI/mL for positivity) and rheumatoid factor IgM (RF) by Elisa (Euro immune cutoff value 20UI/mL for positivity). Serum samples were processed in initial dilution of 1: 101 for the detection of anti CCP_2_ and 1/201 of RF. All tests were performed at the autoimmunity laboratory of the National Institute of Hygiene (Rabat, Morocco) using an automated system (Biorad system PhD™).

**Ethics approval and consent to participate:** the protocol study was reviewed and approved by local institutional review boards and the national ethics committee: ethics committee for biomedical research Mohammed V University- Rabat faculty of medicine and pharmacy of Rabat. The committee's reference number: 70/17.

**Statistical analysis:** statistical analysis was performed using the STATISTICA Stat Soft 12.0 Software (Tulsa, Oklahoma, USA) [15]. Results were given as mean ± standard deviation (S.D.). Pearson χ2 was used to compare categorical variables. Prevalence ratio (PR) and odds ratio (OR) was used for the assessment of risk factors. P-values less than α = 0.05 were considered as significant.

## Results

**General characteristics of our study population:** among the 100 RA patients (83 women and 17 men). The mean age was 57.06 ± 12.21 years with a female predominance of 83%, the mean BMI was 27.14 ± 5.05. The mean duration of illness was 12.19 ± 8.32. Most of the patients had severe activity in moderation with a mean DAS28 3.67 ± 1.77 and HAQ 1.58 ± 0.64 mutations.

**Frequency of *MEFV* gene mutations:** for testing the frequency of *MEFV* gene mutations, we conducted a case-control study involving 100 RA cases and 200 controls. Molecular analyses with Sanger sequencing of the 2 and 10 exons of the *MEFV* gene in our subjects detected 13 missense variants already reported in the literature. We detected three variants in exon 2 (23%) and ten variants in exon 10 (77%). All these variants were predicted by SIFT and polyphen sites (Table 2). All missense mutations previously described in the literature as pathogenic or probably pathogenic. We have eliminated all synonymous variants or variants with a high percentage in the general population. No codon-stop mutation was detected in our cohort. The frequencies of variants detected in our patients were estimated by 24% with 12 variants. The higher frequency was detected in two variants (G222A and M694V) with 5%. Also, we detected in the control group frequency of 2% with only three variants and one of them (S154T) only exist in CG (Table 3).

**Table 2:** detected variants in all subjects (RA patients and control group)

Gene ID	Chromosome	Position	Exon	DNA change	Protein change	Mutation type	dbSNP	Sift	Polyphen
*MEFV*	16	3254607	2	c.461C>G	p.Ser154Trp	Missense	1389511101	0	0.893
*MEFV*	16	3254607	2	c.664G>A	p.Gly222Arg	Missense	772938936	0.01	0.991
*MEFV*	16	3254607	2	c.688G>C	p.Glu230Lys	Missense	104895080	0.02	0.909
*MEFV*	16	3254607	10	c.1832T>A	p.Leu611His	Missense	759706563	0	1
*MEFV*	16	3254607	10	c.2084A>G	p.Lys695Arg	Missense	104895094	-	-
*MEFV*	16	3243407	10	c.2080A>G	p.Met694Val	Missence	61752717	0.16	0.06
*MEFV*	16	3254607	10	c.2160C>G	p.Ile720Met	Missense	104895102	0	1
*MEFV*	16	3254607	10	c.2210G>T	p.Arg737Lys	Missense	139092123	0	0.751
*MEFV*	16	3254607	10	c.2272C>T	p.Pro758Ser	Missense	104895114	0.01	1
*MEFV*	16	3254607	10	c.2126T>G	p.Leu709Arg	Missense	104895184	0.03	0.997
*MEFV*	16	3254607	10	c.2194T>G	Tyr732Asp	Missense	1245305931	0	1
*MEFV*	16	3254607	10	c.2060G>A	p.Gly687Asp	Missense	387907570	0	1
*MEFV*	16	3254607	10	c.2229C>T	p.Phe743Leu	Missense	104895152	-	-

***MEFV*:** Mediterranean fever, **RA:** rheumatoid arthritis, **CG:** control group, **P:** protein

**Table 3:** distribution of *MEFV* gene mutations in the RA and control groups

*MEFV* mutation		RA (n= 100) n (%)	CG (n=200) n (%)
c.461C>G	S154T	-	1 (0.5%)
c.664G>A	G222A	5 (5%)	-
c.688G>C	G230L	4 (4%)	-
c.1832T>A	L611H	1 (1%)	-
c.2080A>G	L695A	4 (4%)	2 (1%)
c.2084A>G	M694V	5 (5%)	1 (0.5%)
c.2160C>G	I720M	1 (1%)	-
c.2210G>T	A737L	1 (1%)	-
c.2272C>T	P758S	2 (2%)	-
c.2126T>G	L709A	2 (2%)	-
c.2194T>G	T732A	1 (1%)	-
c.2060G>A	G687A	2 (2%)	-
c.2229C>T	P743L	1 (1%)	-
**TOTAL**		24 (24%)	4 (2%)

*MEFV*: Mediterranean fever; RA: rheumatoid arthritis; CG: control group

**Immunological profile:** in the immunological profile, the detection of APCA was present 14% in RA patients carrying the *MEFV* mutation and for RF was present 19% in RA patients carrying the *MEFV* mutation. Table 4 shows the correlation between clinical and demographic characteristics and the *MEFV* mutation gene in Moroccan RA patients. We observed that no man has presented a mutation. In addition, BMI was slightly higher in the mutation carrier group than the non-carrier group. We also mentioned in clinical characteristics that HAQ and level of C-reactive protein (CRP) are slightly elevated in the mutation carriers' group.

**Table 4:** association between clinical and demographic characteristics and *MEFV* mutation gene in Moroccan RA patients

		RA patient					
Variables	Carrier (n = 24)	Non-carrier (n = 76)	Total (n = 100)	Prevalence ratio	Odds ratio	X^2^	P-value
Gender(male/female)	24 (0/24)	76 (17/59)	100 (17/83)	0.00	0.00	6.47	0.01*
Age	57.82 ± 11.2	56.86 ± 12.93	57.06 ± 12.56	0.56	0.48	0.84	0.36
BMI	29.18 ± 4.59	26.64 ± 5.05	27.14 ± 5.05	0.24	0.18	8.13	< 0.01*
Inbred marriage	4	19	23	0.67	0.60	0.72	0.4
Smoking	1	13	14	0.27	0.21	2.54	0.11
Alcohol	2	11	13	0.61	0.54	0.61	0.44
Depression	2	6	8	1.05	1.06	0.01	0.94
Familial history	8	29	37	0.85	0.81	0.18	0.67
The duration of the evolution of RA	11.82 ± 6.30	12.28 ± 8.79	12.19 ± 8.32	1.11	1.15	0.08	0.77
HAQ	1.61 ± 0.70	1.57 ± 0.62	1.58 ± 0.64	-	-	-	-
DAS 28 (CRP)	1.96 ± 3.78	3.65 ± 1.78	3.67 ± 1.77	-	-	-	-
ESR	34.1 ± 26	34.33 ± 24.94	34.29 ± 25.02	-	-	-	-
CRP	27.38 ± 29.9	22.57 ± 28.44	23.55 ± 28.65	-	-	-	-
ACPA or anti-CCP_2_	14	64	78	0.39	0.26	7.12	< 0.01*
RF	19	74	93	0.29	0.10	9.28	< 0.01*

**MEFV:** Mediterranean fever, **RA:** rheumatoid arthritis, **CG:** control group, **BMI:** body mass index, **HAQ:** health assessment questionnaire, **ESR:** erythrocyte sedimentation rate, **CRP:** C-reactive protein, **DAS-28:** disease activity score-28, **anti-CCP_2_:** anti-cyclic citrullinated peptide, **RF:** rheumatoid factor. Data are given as mean ± Standard Deviation (S.D.)

**Correlation of the characteristics of patients with RA with *MEFV* mutations:** we found that the prevalence ratio, odds ratio and Pearson X^2^ were higher and present a positive correlation for gender, BMI, smoking, positive RF and positive ACPA between RA patients *MEFV* mutations carrier and non-carrier. We also detected a significant correlation in four characteristics: gender, BMI, positive RF and positive ACPA (p<0.01). No other significant differences were observed between patients with *MEFV* mutations and those without *MEFV*.

## Discussion

This study is the first evaluation of polymorphisms and *MEFV* gene mutations in Morocco with a clinical diagnosis of RA. In the present study, we investigated genetic variations in the *MEFV* gene in healthy Moroccan subjects and patients with RA without the episodic manifestations consistent with FMF. Our results suggest that Mutation analysis of the *MEFV* gene in this cohort of RA patients showed a high frequency of mutations in RA and also an association between clinical and demographic characteristics and the *MEFV* mutation gene.

These *MEFV* mutations have already been reported by some studies such as Migita *et al*. demonstrated that *MEFV* mutations are not rare in RA and that the allele frequencies of R408Q, P369S, E148Q, L110P mutations were 5.6%, 6.7%, 24.2%, 9.5% respectively in patients with RA and that the mutation rate was comparable between RA and healthy controls and suggest that *MEFV* mutations may not be a genetic factor affecting susceptibility to RA [16]. On the other hand, another study in Turkey also reported the high presence of *MEFV* mutations (E148Q, M694V, M694I, M680I, V726A, A744S, R761H, and P369S) and that the carrier rates of these *MEFV* mutations were 25.2% in RA and 23.3% in CG and suggest that mutations in the *MEFV* gene appear to be an aggravating factor in the severity of RA [17]. Although most of these reported mutations were not detected in our study. A single mutation of the *MEFV* gene (M694V) which is common to previous case-control studies [17-21].

Regarding, our study found that the rates of *MEFV* mutation carriers were 24/100 (24%) and 4/200 (2%) in the RA and control group, respectively. The most frequent mutations of the *MEFV* gene were G222A (5%), M694V (5%), L695A (4%) and G230L (4%) in the RA. Exon 2 showed two mutations specifically in RA patients and one mutation in the control group. In addition, exon 10 showed eight mutations significantly correlated with RA. While the M694V and L695A mutations were found to be significantly more frequent in RA patients than in the control group. The mutations M694V observed in Moroccan FMF patients are in exon 10 (M694V) [22]. However, arthritis is a common manifestation of FMF, particularly in M694V homozygotes [23]. The frequency of arthritis in FMF varies between 21% and 77% according to ethnic groups [24]. In addition, the Moroccan population is considered among the subjects from populations at risk with a genotype suggestive of FMF. For this, a mutation of the *MEFV* gene is almost always found in the Moroccan population, such as the M694V variant [22].

In our study, the rates of *MEFV* mutation carriers in PR and GC were not similar. The carrier mutation in the RA group had severe clinical and the level of CRP was higher than in non-carrier patients. We suggest that *MEFV* mutation may indeed be conferring a heightened inflammation as suggested by the increased frequency in inflammatory symptoms [17, 25]. The carrier status for *MEFV* mutations seems to be unique, in that they cause an alteration in the state of “health” [26]. We also studied the presence of a genotype-phenotype potential relationship in RA patients with *MEFV* mutations and those without *MEFV* mutations. This study suggests a strong association between the severity of RA and the presence of mutations in the *MEFV* gene in RA patients. Specifically, a correlation with four known predictors of RA severity: gender, BMI, positive RF and positive ACPA revealed a significant association in RA patients with *MEFV* mutations. These two criteria allow identifying heterogeneity and complexity within this disease [27]. Another study found a significant association between the presence of MEFV mutation and the presence of positive RF in a cohort of Israeli RA patients [13, 28]. As mentioned previously, the positivity of ACPA has been reported as a risk factor for RA. Therefore, our results suggest a role for the *MEFV* gene in the deregulated inflammatory process of RA. Genetic mutations in the Mediterranean fever (*MEFV*) gene, coding for pyrin, are known to influence the severity of RA, but the underlying mechanisms are not fully understood. Anti-citrullinated protein antibodies (ACPAs or anti CCP_2_) are highly specific serological biomarkers [29], that predict the development of more aggressive RA, extra-articular manifestations, and therapeutic response [30]. Therefore, it may be suggested that the *MEFV* gene mutations might not be a susceptibility factor for RA, however, they appear to be an aggravating factor for the severity of RA and consequently, patients with RA might be screened for *MEFV* gene mutations in countries where FMF is frequent, as found in our study and suggested in other studies [13, 18, 25, 31].

Moreover, we found that all men don't present *MEFV* mutations in this research. That proves the high and significant correlation with gender in RA patients. This can be explained by the deregulation of hormones in women might justify why women are much more likely to develop RA than men. When considering gender differences, one has to take into account that the measures of disease activity themselves can be influenced by gender [32]. The other demographic and clinical characteristics including age, inbred marriage, smoking, familial history, alcohol, depression and the duration of the evolution of RA does not present a significant correlation between *MEFV* mutations and patients with RA disease.

This study revealed a new mutation in the *MEFV* gene compared to previous studies. Since RA is genetically a very complex disease that may be due to many other environmental and genetic factors. Sometimes anti-TNF therapy or other non-biological disease-modifying anti-rheumatic drugs (DMARDs) used to treat the symptoms of RA can mask the true clinical expressions of FMF. Also, a patient's phenotype may differ depending on the nature of the FMF mutation, its location, and the presence of other potential genes and environmental modifiers. Further research is needed to establish the specific role [27]. Our results add valuable information to the current knowledge relating to the pathophysiological categorization of autoinflammatory and autoimmune diseases. Recent observations underscore the importance and relevance of an “auto-inflammatory-auto-immune continuum” indicating the close interconnection between innate and adaptive immune mechanisms [33, 34]. A better understanding of molecular pathomechanisms in autoimmune-inflammatory disorder and the relative contribution of innate and adaptive mechanisms will help to introduce individualized and target-directed treatments with increased efficacy and reduced side effects.

This study has certain limitations. The first is that our study cohort is not large enough. Second, we didn't have the means to sequence the entire *MEFV* gene, (we only sequenced the hot spots), nor to carry out a next-generation sequencing to reveal new genes involved in RA. The strengths of this study were the first combination study between genetics and autoimmunity to patients with PR in Morocco and the second, our study combines patients from all regions of Morocco.

## Conclusion

This study suggests that *MEFV* gene mutations maybe not be a susceptibility factor for the development of RA but might increase the severity of RA and consequently, patients with RA might be screened for *MEFV* gene mutations in countries where FMF is frequent. Further research with a larger sample size is needed to determine the actual pathogenic role of *MEFV* mutations in this disease.

### What is known about this topic

- *Several studies have identified that the MEFV gene mutations appear to be an aggravating factor in the severity of RA*.
- *The identification of MEFV gene mutations contributed to a deeper understanding of the pathophysiology of various autoimmune-inflammatory disorders*.

### What this study adds

- *This study suggests that MEFV gene mutations may not be a susceptibility factor for the development of RA but might increase the severity of RA*.
- *This study will enrich the Moroccan database concerning frequencies of the MEFV gene mutations in RA*.
